# Circulating fibroblast growth factor 23 levels and incident dementia: The Framingham heart study

**DOI:** 10.1371/journal.pone.0213321

**Published:** 2019-03-04

**Authors:** Emer R. McGrath, Jayandra J. Himali, Daniel Levy, Sarah C. Conner, Matthew P. Pase, Carmela R. Abraham, Paul Courchesne, Claudia L. Satizabal, Ramachandran S. Vasan, Alexa S. Beiser, Sudha Seshadri

**Affiliations:** 1 Department of Neurology, Brigham & Women’s Hospital, Boston, MA, United States of America; 2 Harvard Medical School, Boston, MA, United States of America; 3 Framingham Heart Study, Framingham, MA, United States of America; 4 Boston University School of Public Health, Boston, MA, United States of America; 5 Boston University School of Medicine, Boston, MA, United States of America; 6 Population Sciences Branch, National Heart, Lung and Blood Institutes of Health, Bethesda, MD, United States of America; 7 Melbourne Dementia Research Centre, The Florey Institute for Neuroscience and Mental Health, Victoria, Australia; 8 Glenn Biggs Institute for Alzheimer’s & Neurodegenerative Diseases, University of Texas Health Sciences Center, San Antonio, TX, United States of America; Nathan S Kline Institute, UNITED STATES

## Abstract

**Background:**

Fibroblast growth factor 23 is an emerging vascular biomarker, recently associated with cerebral small vessel disease and poor cognition in patients on dialysis. It also interacts with klotho, an anti-aging and cognition enhancing protein.

**Objective:**

To determine if circulating Fibroblast growth factor 23 (FGF23) is associated with new-onset cognitive outcomes in a community-based cohort of cognitively healthy adults with long-term follow-up.

**Methods:**

We measured serum FGF23 levels in 1537 [53% women, mean age 68.7 (SD 5.7)] dementia-free Framingham Offspring participants at their 7^th^ quadrennial examination (1998–2001), and followed these participants for the development of clinical all-cause dementia and Alzheimer’s disease (AD). Secondary outcomes included MRI-based structural brain measures, and neurocognitive test performance at exam 7.

**Results:**

During a median (Q1, Q3) 12-year (7.0, 13.3) follow up, 122 (7.9%) participants developed dementia, of whom 91 (5.9%) had AD. Proportional-hazards regression analysis, adjusted for age, sex, education, systolic blood pressure, antihypertensive medication, prevalent cardiovascular disease, diabetes mellitus, smoking status and apoE ε4 carrier status, revealed that higher serum FGF23 levels were associated with an increased risk of incident dementia and AD (Hazard ratio [HR] per 1 standard deviation increment in inverse transformed FGF23 level 1.25, 95% CI 1.02–1.53, and 1.32, 95% CI 1.04–1.69, respectively). There was no significant interaction according to presence/absence of significant renal impairment (eGFR <30 versus ≥30ml/min) and risk of dementia (based on 1537; p = 0.97).

**Conclusions:**

Higher circulating FGF23 is associated with an increased risk of dementia, suggesting that FGF23-related biological pathways may play a role in the development of dementia.

## Introduction

Dementia is a major health problem. An estimated 35 million people worldwide have dementia and this number is expected to triple over the next three decades due to an aging population[1]. Genetic factors, such as the apoE ε4 allele, and vascular risk factors, such as hypertension, can explain a proportion of dementia risk. However, a large proportion of dementia risk remains unexplained, necessitating the identification of additional underlying pathways and newer biomarkers for dementia.

Fibroblast growth factor 23 (FGF23) is a hormone secreted by osteocytes which regulates phosphate homeostasis and transport in the kidney and vitamin D metabolism, through lowering serum phosphate and vitamin D levels[2]. FGF23 has emerged as a novel biomarker for vascular disease including stroke[3]. In addition, higher FGF23 levels have been associated cross-sectionally with radiographic markers of cerebral small vessel disease[4] as well as with poor cognitive performance in a small number of patients on hemodialysis[5]. However, it is unknown if serum FGF23 levels are associated with cognitive performance and late-life dementia in the general population. Discovering novel biomarkers for dementia can enable earlier identification of individuals at increased risk, further our understanding of the biological pathways underlying dementia, and potentially identify new therapeutic targets and opportunities for intervention.

In the present study, we determined the association between serum FGF23 levels and cognitive performance, structural MRI brain measures predictive of dementia, and clinically confirmed, new-onset all-cause dementia and Alzheimer’s disease (AD), in a large, community-based, prospective cohort.

## Methods

### Study sample

**Fig 1:**
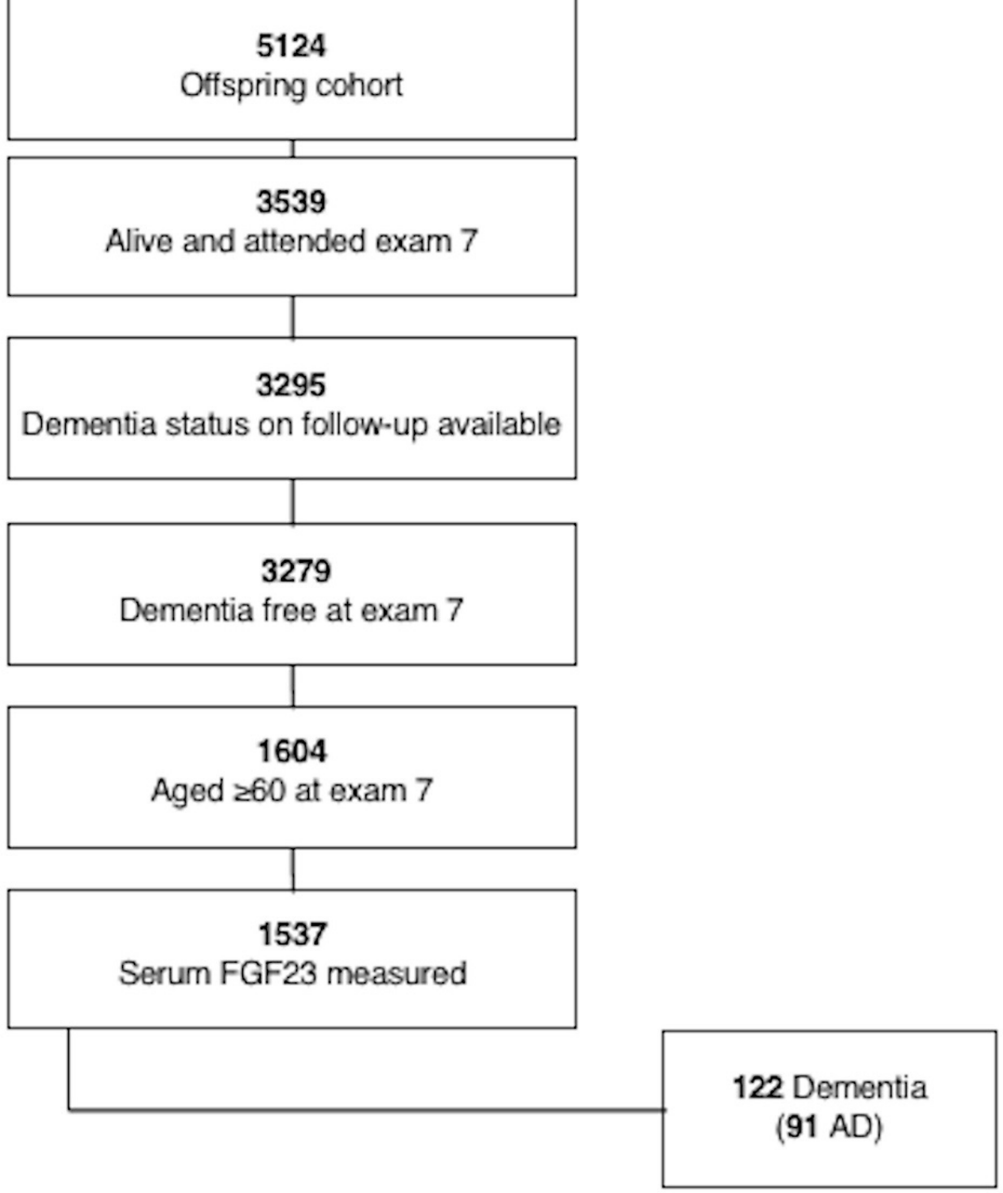
Flow of cohort participants.

The Framingham Offspring Study (recruited 1971–1975) is a community-based cohort and has been longitudinally followed for cardiovascular risk factors and occurrence of cognitive decline, dementia and vascular outcomes for more than 40 years.[6] Participants are examined quadrennially from date of entry into the cohort. We included Framingham Offspring cohort participants who attended examination 7 (1998–2001), had serum biomarkers measured and were free of a diagnosis of dementia at this examination, and had subsequent follow-up for incident dementia. For the cognitive performance outcomes, we included those individuals who participated in cognitive testing at examination 7. All participants provided written informed consent at each examination cycle. The study protocols and consent forms were approved by the institutional review board at the Boston University Medical Center. The flow of cohort participants is shown in **Fig 1**.

### Outcome measures

The primary outcome measure was incident all-cause dementia developing at any time after the seventh Offspring examination visit. Starting from the fifth examination, participants are systematically screened for the development of dementia at each visit using the Mini-Mental State Examination (MMSE), as well as annual health status updates. From examination seven, all participants are invited to complete neuropsychological testing and a brain MRI. If any concern for impaired cognition is raised by a participant, family member, or Framingham study physician, or the Mini-Mental State Examination (MMSE) score is below the education-based cutoff, three points lower than the preceding examination, or five points lower than the participant’s highest recorded score, more detailed cognitive testing is performed.[7] Participants with suspected cognitive impairment who do not meet diagnostic criteria for dementia undergo additional yearly neuropsychological and neurological assessments between the scheduled Offspring examinations. Dementia was diagnosed according to the criteria of the Diagnostic and Statistical Manual of Mental Disorders, 4th edition, requiring impairment in memory and at least one other domain of cognitive function, along with documented functional disability. The final diagnosis and date of diagnosis of dementia were based on a review of all available neurological examination records, neuropsychological assessments, study records, hospital, nursing home and outpatient clinic records, neuro-imaging results, family interview information, and autopsy results (when available) by a committee that included at least one neurologist and one neuropsychologist. Clinically confirmed AD was included as a secondary outcome measure and was diagnosed when subjects met the criteria of the National Institute of Neurological and Communicative Disorders and Stroke and the Alzheimer’s Disease and Related Disorders Association for definite, probable, or possible Alzheimer’s disease.[8] Further details have previously been published.[9]

Additional secondary outcomes included performance on selected neuropsychological tests (n = 2230), including the Trail Making Test B minus A (executive function, processing speed), Hooper Visual Organization Test (visual processing), Visual Reproductions delayed recall (visual memory), Similarities (verbal reasoning and categorization) and Logical Memory delayed recall (verbal memory), to determine if serum FGF23 was cross-sectionally associated with deficits in particular cognitive domains. We also evaluated a weighted global cognitive test score as an overall measure of cognition. This outcome variable was created using principal component analysis and forcing a single score solution. This score combined weighted loadings for the individual cognitive test components described above, with higher scores indicating superior cognitive performance for each individual component, with the exception of the Trail Making test, whereby higher scores indicate poorer cognitive performance. Details on neurocognitive testing in the Framingham Offspring cohort have previously been published[10].

Other secondary outcomes included structural MRI brain measures (n = 1976), including presence of covert brain infarcts [(CBI) determined manually by an operator based on size ≥3mm, location, and imaging characteristics of the lesion using previously described methods[11]], total cerebral brain volume [(TCBV) defined as supratentorial brain volume as a percentage of the intracranial volume determined from coronal sections on quantitative MRI using previously described methods[12]], hippocampal volume (HV) and white matter hyperintensity volume [(WMHV) determined according to previously published methods with documented high reliability[11, 13, 14]]. Imaging data was centrally processed and analyzed by operators blinded to participant identity, age, sex, vascular risk factors and cognitive performance on neuropsychological testing. All imaging analyses were performed using the image analysis package, QUANTA 6.2, operating on a Sun Micro-systems (Santa Clara, CA) Ultra 5 workstation.

### Laboratory measurements of FGF23

Fasting early morning serum samples were drawn from the antecubital vein of participants who had been lying supine for ten minutes. Samples were immediately centrifuged and stored at -80°C, remaining frozen until the time of the assay. Serum FGF23 concentrations were measured using a multiplex immunoassay. Lower and upper detection limits were 1.87E+00 and 5.86E+04 pg/mL, respectively. The inter-assay coefficient of variation ranged from 5.49% to 5.68%.

### Covariates

We adjusted for baseline covariates, measured at examination 7, associated with an increased risk of dementia, including age, sex, education (self-reported and categorized as: no high school degree, high school degree but no college degree, some years of college, and college degree or higher), systolic blood pressure, use of antihypertensive medication, prevalent cardiovascular disease [(CVD) coronary artery disease including angina, coronary insufficiency, myocardial infarction; peripheral vascular disease including intermittent claudication; cerebrovascular disease including stroke and transient ischemic attack; and congestive heart failure], diabetes mellitus, current smoking, apolipoprotein E ε4 (ApoE ε4) carrier status, estimated glomerular filtration rate (eGFR) and serum 25-hydroxy vitamin D (as a marker of mineral metabolism). Serum 25-hydroxy vitamin D was measured using the Cobra II Auto-Gamma assay, with a measuring range from 5.0 to 100.0 ng/ml. Measurements were made in duplicate. The inter-assay coefficient of variation ranged from 8.5% to 13.2%. Fasting early morning serum samples were drawn and stored at -80°C, remaining frozen until the time of the assay. We also adjusted for age squared (for MRI-based outcomes given that the association between age and brain volume is non-linear) and time between measurement of serum FGF23 levels and MRI (for MRI-based outcomes) and time between measurement of serum FGF23 levels and neurocognitive testing (for neurocognitive outcomes).

### Statistical analysis

To evaluate FGF23 as a continuous variable, we inverse transformed FGF23 values to normalize the distribution. These values were then multiplied by -1 to restore directionality, and were standardized. We also calculated quartiles of serum FGF23 levels, and compared quartiles 2–4 to the lowest quartile. We utilized multivariable-adjusted Cox proportional hazards models to examine the association between each of serum FGF23 levels (inverse transformed, directionality adjusted and standardized) (primary analysis) and FGF23 quartiles and risk of incident dementia and AD (secondary analysis). Follow-up for dementia was from the baseline exam to the time of the incident event. For persons without incident events, follow-up was up to the time of death or the date the participant was last known to be event free. The proportionality of hazards assumption was upheld, and results were expressed as hazard ratios (HR) and 95% confidence intervals (CIs) with p-values. Model 1 adjusted for age and sex; model 2 (primary analysis model) additionally adjusted for education, CVD history, systolic blood pressure, use of antihypertensive medication, diabetes mellitus, current smoking status and ApoE ε4 carrier status; model 3 additionally adjusted for eGFR and serum vitamin D (as a marker of mineral metabolism). We evaluated model discrimination (C-statistic[15] and integrated discrimination improvement [IDI][16, 17]), and improvement in risk prediction (net reclassification improvement [NRI] index[16, 18]) for incident dementia and AD, following the addition of FGF23 to the model.

We used linear regression models to examine the cross-sectional association between FGF23 levels and neuropsychological test performance, adjusting for the covariates mentioned above. We also used a series of linear and logistic regression models to examine the cross-sectional association between serum FGF23 levels and MRI-based structural brain measures, including CBI, WMHV, TCBV and HV. Given FGF23 has previously been associated with poor cognitive outcomes in patients with significant renal impairment, we completed subgroup analysis according to age (aged <median of 68 years versus ≥68 years), sex and presence/absence of renal impairment (defined as eGFR <60 versus ≥60ml/min and eGFR <30 versus ≥30ml/min [indicating more severe renal impairment]), to determine if FGF23 was also associated with cognitive outcomes in patients without significant renal disease. Finally we completed a sensitivity analyses excluding individuals with a history of prior stroke (n = 1496).

All results were considered significant if p < 0.05, except for tests of interaction which were considered significant if p < 0.10. Analyses were conducted using SAS statistical software, v9.4 (SAS Institute Inc., Cary, NC).

#### Data availability statement

The data used in these analyses can be obtained from the NHLBI and the NCBI dbGaP.

## Results

The study cohort included 1537 participants for the primary outcome of incident dementia. During a median (Q1, Q3) 12 (7.0, 13.3) year follow up, 122 (7.9%) participants were diagnosed with dementia, 91 of whom were diagnosed with clinical AD. 74 (4.8%) of individuals died from cardiovascular disease during follow-up. The mean age of participants at baseline was 68.7 years (SD 5.7) and 53% were women. Baseline characteristics are shown in **Table 1**. Patients in the top quartile of FGF23, compared to the bottom quartile, were more likely to be female, have a history of diabetes, prior CVD or a history of atrial fibrillation. The majority of cohort participants did not have significant renal impairment at baseline (99%) (**Table 1**).

**Table 1:** Baseline characteristics of dementia cohort.

Variable	Overall (n = 1537)	FGF23 Q1 (n = 384)	FGF23 Q2 (n = 384)	FGF23 Q3 (n = 384)	FGF23 Q4 (n = 385)
No. (%)					
Age, y, mean (SD)	68.7±5.7	68.4±5.7	68.2±5.5	69.3±5.8	68.9±5.8
Women	815 (53.0)	175 (49.5)	190 (49.5)	222 (57.8)	228 (59.2)
Systolic blood pressure, mmHg, mean (SD)	132±19	133±18	131±19	133±20	133±20
BMI, kg/m^2^, median (Q1, Q3)	27.4 (24.7,30.1)	27.1 (24.4,30.7)	27.4 (24.7,30.6)	27.6 (24.8,31.2)	27.5 (24.8,30.9)
FGF23, pg/ml, median (Q1, Q3)	48.4 (37.3, 68.5)	30.4 (26.4, 33.9)	42.8 (40.3, 44.9)	56.7 (51.9, 62.1)	103.0 (79.0, 160.0)
eGFR <30, ml/min	9 (0.6)	0 (0.0)	0 (0.0)	3 (0.8)	6 (1.6)
eGFR, ml/min, mean (SD)	75.3±15.1	79.3±12.2	77.0±14.2	73.5±15.0	71.4±17.4
Vitamin D, ng/ml	20.0±7.5	19.0±6.5	20.2±8.0	20.0±7.7	20.6±7.6
Education					
No high school degree	97 (6.5)	26 (7.0)	18 (4.8)	26 (7.0)	27 (7.2)
High school degree	508 (34.0)	112 (30.0)	145 (38.8)	126 (33.8)	125 (33.5)
Some years of college	430 (28.8)	111 (29.7)	97 (25.9)	120 (32.2)	102 (27.4)
College degree	459 (30.7)	125 (33.4)	114 (30.5)	101 (27.1)	119 (31.9)
Anti-hypertensive medication	678 (44.1)	163 (42.5)	167 (43.5)	166 (43.2)	182 (47.4)
Current smoker	129 (8.4)	27 (7.0)	31 (8.1)	33 (8.6)	38 (9.9)
Diabetes	253 (16.7)	53 (14.0)	56 (14.9)	70 (18.4)	74 (19.4)
ApoE E4 allele	347 (22.9)	85 (22.5)	106 (27.9)	82 (21.6)	74 (19.6)
Prevalent CVD	293 (19.1)	57 (14.8)	69 (18.0)	77 (20.1)	90 (23.4)
Atrial fibrillation	95 (6.2)	14 (3.7)	20 (5.2)	30 (7.8)	31 (8.1)
Stroke	41 (2.7)	9 (2.3)	7 (1.8)	11 (2.9)	14 (3.6)

Abbreviations: SD, standard deviation; CVD, cardiovascular disease; APOE E4, apolipoprotein E4 allele.

Baseline demographic and clinical characteristics were defined at examination 7.

694 individuals had missing data for serum vitamin D levels

### FGF23 and dementia

**Fig 2:**
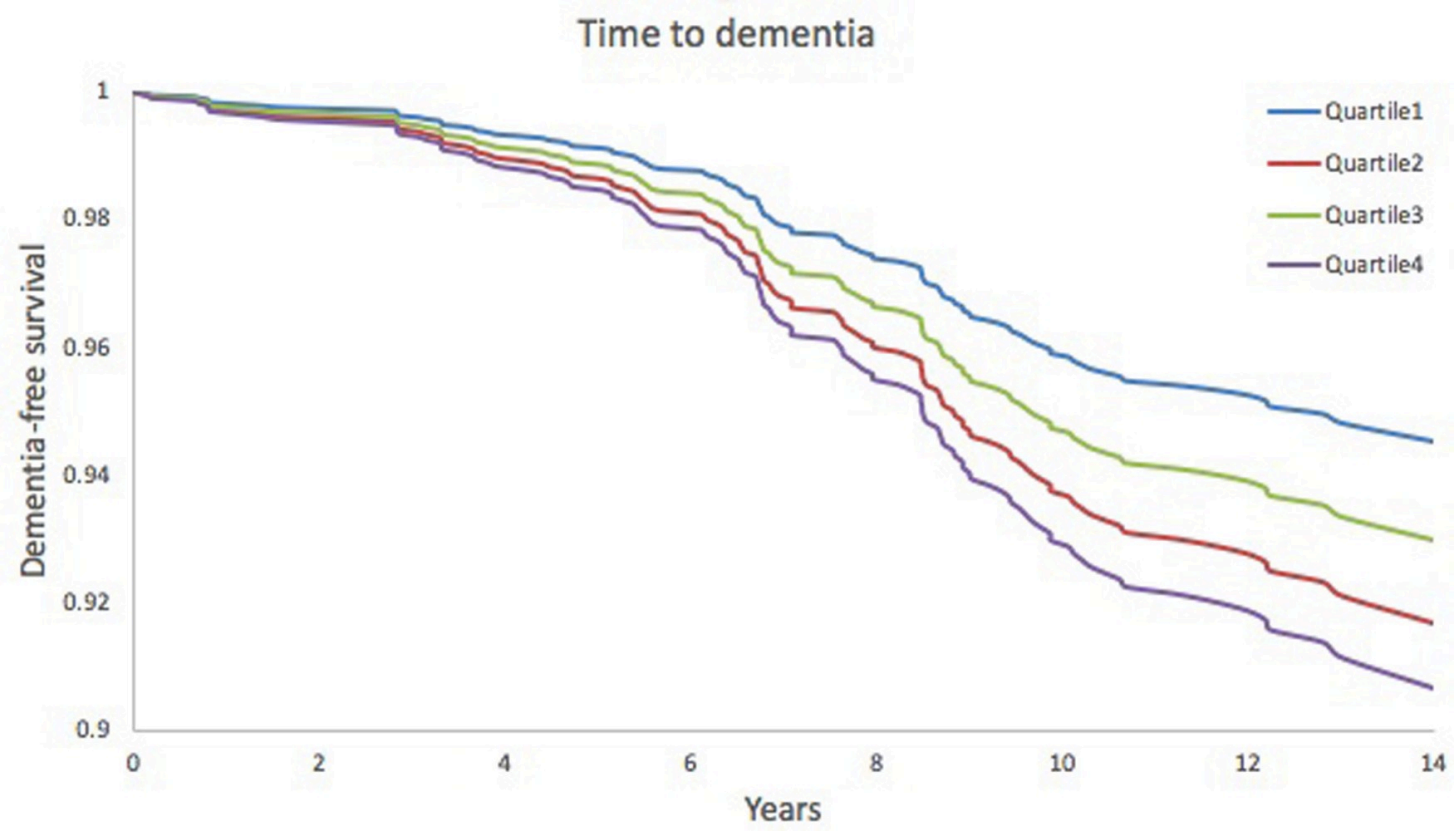
Kaplan Meier plot—time to dementia. Adjusted for age, sex, education, systolic blood pressure, use of antihypertensive medication, diabetes mellitus, current smoking status, prevalent cardiovascular disease and ApoE ε4 carrier status.

**Fig 3:**
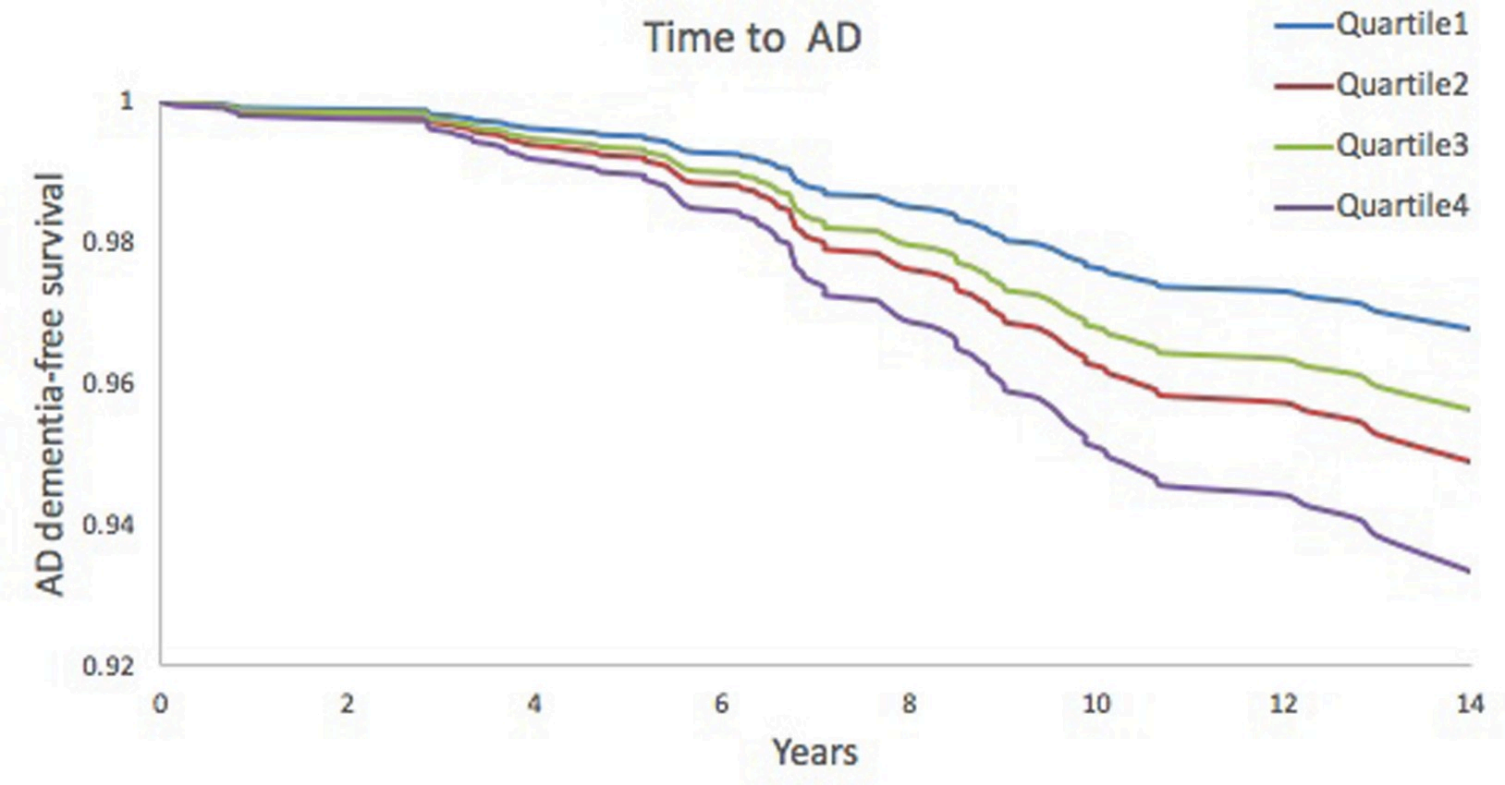
Kaplan-Meier plot—time to Alzheimer’s disease dementia. Adjusted for age, sex, education, systolic blood pressure, use of antihypertensive medication, diabetes mellitus, current smoking status, prevalent cardiovascular disease and ApoE ε4 carrier status.

On multivariable Cox proportional-hazards analysis adjusted for conventional risk factors (age, sex, education, systolic blood pressure, use of antihypertensive medications, prevalent cardiovascular disease and apoE ε4 carrier status), higher serum FGF23 was associated with an increased risk of incident dementia (HR 1.25, 95% CI 1.02–1.53) and AD (HR 1.32, 95% CI 1.04–1.69) per 1 standard deviation unit increment in inverse transformed value. (**Table 2**). A test for linear trends across quartiles of FGF23 was significant for dementia (HR 1.20, 95% CI 1.02–1.41) and AD (HR 1.28, 95% CI 1.06–1.55). On multivariable analysis, the highest quartile of FGF23, compared to the lowest quartile, was also associated with an increased risk of dementia (HR 1.75, 95% CI 1.01–3.03) and AD (HR 2.10, 95% CI 1.09–4.07). **Figs 2 and 3**show time to onset of dementia and AD dementia, respectively, according to FGF23 quartiles.

**Table 2:** FGF23 and risk of incident dementia and AD.

Biomarker		Dementia		Alzheimer’s disease	
FGF23	Model	HR (95% CI)	P-value	HR (95% CI)	P-value
Per SDU increase	1	1.28 (1.05–1.56)	0.01	1.37 (1.08–1.73)	<0.01
	2	1.25 (1.02–1.53)	0.04	1.32 (1.04–1.69)	0.03
	3	1.30 (0.99–1.71)	0.06	1.24 (0.90–1.72)	0.19
Q1	1	Ref.		Ref.	
Q2		1.60 (0.91–2.83)	0.10	1.70 (0.85–3.40)	0.13
Q3		1.50 (0.86–2.61)	0.16	1.68 (0.86–3.29)	0.13
Q4		1.93 (1.12–3.32)	0.02	2.37 (1.24–4.53)	<0.01
Q1	2	Ref.		Ref.	
Q2		1.55 (0.87–2.74)	0.13	1.59 (0.79–3.20)	0.19
Q3		1.29 (0.73–2.30)	0.38	1.36 (0.68–2.73)	0.18
Q4		1.75 (1.01–3.03)	0.047	2.10 (1.09–4.07)	0.03
Q1	3	Ref.		Ref.	
Q2		1.46 (0.67–3.18)	0.34	1.41 (0.57–3.52)	0.46
Q3		1.19 (0.54–2.63)	0.67	1.19 (0.47–2.98)	0.72
Q4		1.65 (0.78–3.48)	0.19	1.68 (0.70–4.02)	0.24

Abbreviations: FGF23, Fibroblast Growth Factor 23; HR, hazard ratio; CI, confidence interval; Ref, reference.

Model 1: adjusted for age and sex. Model 2: adjusted for age, sex, education, systolic blood pressure, use of antihypertensive medication, prevalent cardiovascular disease, diabetes mellitus, current smoking and ApoE ε4 carrier status. Model 3: model 2 + additionally adjusted for eGFR and serum vitamin D.

Note, 707 (46%) individuals were excluded in model 3 due to missing data for serum vitamin D and/or eGFR levels

FGF23 was inverse transformed, directionality adjusted and standardized.

FGF23 was assessed according to quartiles.

There was no significant interaction according to presence/absence of significant renal impairment and risk of dementia (eGFR <30 versus ≥30ml/min, p = 0.974; eGFR <60 versus ≥60ml/min, p = 0.149) or sex and risk of dementia (p = 0.67). There was a significant interaction according to age and risk of dementia (p = 0.002; HR 1.14 [95% CI 0.75–1.75] for age <68 years versus HR 1.32 [95% CI 1.06–1.65] for age ≥68 years). On sensitivity analyses, excluding those with prior stroke, results were consistent (**Table 3**).

**Table 3:** FGF23 and risk of incident dementia and AD, excluding those with prior stroke.

Biomarker	Model	Dementia		Alzheimer’s disease	
		HR (95% CI)	p-value	HR (95% CI)	p-value
Per SDU increase	1	1.29 (1.06–1.58)	0.01	1.36 (1.07–1.72)	0.01
	2	1.27 (1.03–1.57)	0.02	1.33 (1.04–1.71)	0.02
	3	1.33 (1.01–1.77)	0.046	1.25 (0.90–1.73)	0.19
Q1	1	Ref		Ref	
Q2		1.39 (0.78–2.49)	0.27	1.54 (0.76–3.11)	0.23
Q3		1.57 (0.90–2.74)	0.12	1.76 (0.90–3.44)	0.10
Q4		1.86 (1.08–3.22)	0.03	2.24 (1.16–4.32)	0.02
Q1	2	Ref		Ref	
Q2		1.34 (0.74–2.41)	0.33	1.44 (0.71–2.93)	0.31
Q3		1.44 (0.82–2.55)	0.21	1.55 (0.78–3.09)	0.21
Q4		1.74 (1.00–3.03)	0.05	2.05 (1.05–4.00)	0.03
Q1	3	Ref		Ref	
Q2		1.14 (0.50–2.56)	0.76	1.25 (0.49–3.16)	0.64
Q3		1.20 (0.54–2.65)	0.65	1.21 (0.48–3.04)	0.68
Q4		1.52 (0.71–3.24)	0.28	1.54 (0.63–3.75)	0.34

Sample size = 1496

Abbreviations: FGF23, Fibroblast Growth Factor 23; HR, hazard ratio; CI, confidence interval.

Model 1: adjusted for age and sex. Model 2: adjusted for age, sex, education, systolic blood pressure, use of antihypertensive medication, prevalent cardiovascular disease, diabetes mellitus, current smoking status and ApoE ε4 carrier status. Model 3: model 2 + additionally adjusted for eGFR and serum vitamin D.

FGF23 was inverse transformed, directionality adjusted and standardized.

### FGF23 and secondary outcomes

Serum FGF23 was not associated with global cognitive test performance at exam 7 (ß±SE, -0.004±0.02, p = 0.81), or with performance on individual neurocognitive tests including the Trail Making Test parts B-A, Hooper visual Organization Test, delayed visual reproduction test, similarities test or delayed logical memory test (**Table 4**). Serum FGF23 was not associated with structural brain measures including total cerebral brain volume (-0.02±0.06, p = 0.74) and white matter hyperintensity volume (-0.02±0.02, p = 0.45) (**Table 5**).

**Table 4:** FGF23 and neuropsychological test performance.

	Global cognition (weighted score units)		Similarities (n correct)		Visual Reproductions (n correct after delay)		Logical Memory Delayed (n correct)		Trail Making B-A (min)^b^		Hooper Visual Organization Test^b^	
	β±SE	p-value	β±SE	p-value	β±SE	p-value	β±SE	p-value	β±SE	p-value	β±SE	p-value
**Model 1**												
SDU^a^	-0.004±0.02	0.81	-0.02±0.07	0.78	-0.03±0.06	0.61	0.05±0.07	0.50	-0.004±0.004	0.30	0.01±0.01	0.35
Q1	Ref											
Q2	-0.035±0.05	0.46	-0.26±0.19	0.17	-0.19±0.18	0.28	0.05±0.20	0.80	-0.003±0.01	0.83	-0.01±0.03	0.80
Q3	-0.00001±0.05	0.99	-0.08±0.19	0.66	0.02±0.18	0.93	0.27±0.20	0.18	-0.015±0.01	0.19	0.003±0.03	0.91
Q4	-0.008±0.05	0.86	-0.11±0.19	0.55	-0.10±0.18	0.59	0.12±0.20	0.55	-0.003±0.01	0.82	0.03±0.03	0.35
**Model 2**												
SDU^a^	-0.001±0.02	0.94	-0.003±0.07	0.96	-0.03±0.07	0.69	0.03 ± 0.07	0.67	-0.004±0.004	0.39	0.01±0.01	0.28
Q1	Ref											
Q2	-0.034±0.05	0.48	-0.28±0.19	0.14	-0.14±0.18	0.45	0.03±0.2	0.87	0.001±0.01	0.91	-0.01±0.03	0.75
Q3	-0.0004±0.05	0.99	-0.06±0.19	0.77	0.01±0.18	0.97	0.22±0.2	0.28	-0.014±0.01	0.23	0.003±0.03	0.92
Q4	-0.004±0.05	0.94	-0.10±0.19	0.61	-0.07±0.18	0.70	0.07±0.2	0.73	-0.0004±0.01	0.98	0.03±0.03	0.30
**Model 3**												
SDU^a^	-0.03±0.02	0.17	-0.05±0.09	0.60	-0.07±0.09	0.40	-0.06±0.1	0.52	-0.006±0.01	0.29	0.007±0.01	0.63
Q1	Ref											
Q2	-0.07±0.06	0.28	-0.57±0.25	0.02	-0.14±0.25	0.58	-0.13±0.27	0.63	-0.003±0.02	0.87	0.008±0.04	0.84
Q3	-0.05±0.06	0.45	-0.24±0.24	0.32	-0.13±0.24	0.59	0.15±0.27	0.58	-0.015±0.02	0.32	0.006±0.04	0.88
Q4	-0.08±0.06	0.22	-0.3±0.25	0.23	-0.07±0.25	0.78	-0.23±0.27	0.40	-0.003±0.02	0.86	0.025±0.04	0.52

Abbreviations: SDU, Standard deviation units; SE, Standard error.

Model 1: adjusted for age, sex, education and time from blood draw to neuropsychological testing.

Model 2: model 1 + additionally adjusted for systolic blood pressure, use of antihypertensive medication, and prevalent cardiovascular disease.

Model 3: model 2 + additionally adjusted for eGFR and serum vitamin D.

^a^Standardized, inverse transformed and directionality-adjusted FGF23.

^b^log transformed.

**Table 5:** FGF23 and MRI markers of structural brain injury.

	Total brain volume (%x100)		Hippocampal volume (%)		WMHV^b^ (%)		Covert brain infarcts (n)	
	β±SE	p-value	β±SE	p-value	β±SE	p-value	OR (95% CI)	p-value
**Model 1**								
SDU^a^	-0.02±0.06	0.74	0.0001±0.001	0.93	-0.02±0.02	0.45	0.93 (0.80, 1.07)	0.30
Q1	Ref							
Q2	0.31±0.18	0.08	-0.003±0.003	0.27	-0.02±0.06	0.77	0.52 (0.33, 0.81)	0.02
Q3	0.07±0.18	0.68	-0.001±0.003	0.62	-0.05±0.06	0.34	0.76 (0.50, 1.13)	0.82
Q4	-0.002±0.18	0.99	-0.001±0.003	0.70	-0.001±0.06	0.99	0.73 (0.49, 1.11)	0.99
**Model 2**								
SDU^a^	0.01±0.06	0.82	0.0003±0.001	0.81	-0.02±0.02	0.45	0.91 (0.78, 1.06)	0.22
Q1	Ref							
Q2	0.35±0.18	0.04	-0.003±0.003	0.29	-0.02±0.06	0.76	0.52 (0.34, 0.82)	0.03
Q3	0.10±0.18	0.56	-0.001±0.003	0.67	-0.05±0.06	0.36	0.76 (0.50, 1.14)	0.78
Q4	0.10±0.18	0.58	-0.001±0.003	0.77	-0.01±0.06	0.93	0.71 (0.47, 1.08)	0.85
**Model 3**								
SDU^a^	-0.02±0.08	0.85	0.001±0.001	0.66	-0.02±0.03	0.55	0.86 (0.71, 1.04)	0.12
Q1	Ref							
Q2	0.44±0.24	0.06	-0.002±0.004	0.57	-0.05±0.08	0.49	0.49 (0.28, 0.88)	0.11
Q3	0.07±0.23	0.77	-0.0001±0.004	0.99	-0.10±0.07	0.19	0.68 (0.40, 1.15)	0.95
Q4	0.02±0.24	0.95	-0.001±0.004	0.80	0.004±0.08	0.96	0.60 (0.35, 1.04)	0.54

Abbreviations: FGF23, Fibroblast growth factor 23; SDU, Standard deviation units; SE, Standard error; WMHV, White matter hyperintensity volume.

Model 1: adjusted for age, age squared, sex and time from blood draw to MRI brain.

Model 2: model 1 + additionally adjusted for systolic blood pressure, use of antihypertensive medication, and prevalent cardiovascular disease.

Model 3: model 2 + additionally adjusted for eGFR and serum vitamin D.

^a^Standardized, inverse transformed and directionality-adjusted FGF23.

^b^log transformed.

### Risk prediction for dementia

The C-statistic for the model of conventional risk factors for dementia was 0.81 (95% CI 0.77–0.84), with no significant change following the addition of FGF23 to the model. In addition, there was no significant improvement in risk prediction following addition of FGF23 to the model (NRI 0.12, 95% CI -0.08–0.33) (**Table 6**).

**Table 6:** Model discrimination and risk reclassification.

	Dementia				Alzheimer’s			
	C-statistic (95% CI)	Relative IDI (95% CI)	Overall NRI (95% CI)	*NRI, events NRI, nonevents	C-statistic (95% CI)	Relative IDI (95% CI)	Overall NRI (95% CI)	*NRI, events NRI, nonevents
Model 2	0.81 (0.77–0.84)	-	-	-	0.84 (0.81–0.88)	-	-	-
Model 2 + FGF23	0.81 (0.77–0.84)	0.03 (-0.004–0.07)	0.12 (-0.08–0.33)	0.11 0.01	0.85 (0.81–0.88)	0.05 (0.003–0.10)	0.21 (-0.02–0.45)	0.17 0.04

Abbreviations: IDI, integrated discrimination improvement; NRI, net reclassification improvement; FGF23, Fibroblast growth factor CI, confidence interval.

Model 2: adjusted for age, sex, education, systolic blood pressure, use of antihypertensive medication, prevalent cardiovascular disease and ApoE4 carrier status.

FGF23 was inverse transformed, directionality adjusted and standardized.

* Proportion of events correctly reclassified

Proportion of non-events correctly reclassified.

## Discussion

In our study, higher serum FGF23 was associated with an increased risk of incident dementia and AD but was not associated with structural brain measures predictive of vascular brain injury or with performance on neurocognitive testing.

We found that FGF23 was associated with an increased risk of dementia and AD. While previous studies have reported an association between serum FGF23 and radiographic markers of cerebral small vessel disease and cognitive performance,[4, 5] there have been no studies to date looking at clinically confirmed dementia or AD. There are a number of possible explanations for the association we observed between FGF23 and dementia. First, higher serum FGF23 may simply be a marker of increased vascular risk. Increased FGF23 levels have cross-sectionally been associated with vascular risk factors, including smoking, waist circumference, history of cardiovascular disease and serum glucose levels[19]. In addition, increased FGF23 levels have also been associated with vascular calcification and left ventricular hypertrophy[20]. In our study, participants in the top quartile of serum FGF23, compared to the bottom quartile, were more likely to have a history of stroke or CVD. However, the association between FGF23 and dementia was not attenuated after adjusting for vascular risk factors, suggesting that the association is unlikely to be solely mediated through increased systemic vascular risk. In addition, we observed no association between FGF23 and structural measures of vascular brain injury, including CBI and WMHV on cross-sectional analysis, although admittedly we were unable to assess for a longitudinal association between serum FGF23 levels and changes in structural MRI brain measures over time. Other studies have similarly reported minimal to no attenuation of the association between FGF23 and risk of cardiovascular outcomes after adjusting for traditional vascular risk factors[3], supporting our findings.

Second, the association may be mediated by intermediate markers of mineral metabolism. FGF23 inhibits 1-alpha hydroxylase activity in the kidney, resulting in lower vitamin D levels, while also acting as a phosphaturic factor, lowering serum phosphate levels[2]. Vitamin D deficiency has been shown to be predictive of cognitive decline in older adults, providing a potential explanation for an association between higher FGF23 levels and dementia[21]. In our cohort, after adjusting for serum vitamin D and eGFR, the association between serum FGF23 and all-cause dementia (HR 1.30, 95% CI 0.99–1.71) was no longer significant, although the change was negligible and likely related to the reduction in sample size (45% of individuals had missing data for serum vitamin D), suggesting that effects on bone mineral metabolism do not appear to mediate its association with poorer cognitive outcomes. Admittedly, serum phosphate, calcium and parathyroid hormone were not measured at examination 7 and so we were unable to adjust for their effects. However, these additional markers would be expected to show collinearity with vitamin D, and so adjusting for vitamin D should largely account for their effects.

Third, FGF23 may increase the risk of dementia directly through its interaction with klotho, an anti-aging protein. While FGF23 is primarily expressed in bone, it is also found in high concentrations in the brain and cerebrospinal fluid, produced in the ventrolateral nucleus of the thalamus [5, 22]. It is reported to affect neuronal morphology and synaptic density[23]. In the kidney, FGF23-mediated activation of FGF receptors requires the co-receptor klotho[24]. Klotho is also found in the choroid plexus in the brain[22] as well as in the brain parenchyma[25] and may play a role in FGF23 activity in the brain. Klotho has been shown to be neuroprotective *in vitro* and *in vivo*, protecting primary neurons from oligomeric amyloid beta (Abeta)[26]. Klotho loss of function mice exhibit features consistent with accelerated aging[25], while transgenic mice with klotho overexpression perform better in tests of memory and learning and live longer compared to control mice[27, 28]. Elevated klotho levels in mice are thought to result in enhanced N-methyl-D-aspartate (NMDA) receptor related functions and postsynaptic enrichment of the GluN2B subunit of the NMDA receptor in the hippocampus and cortex, which plays key roles in **learning** and memory[27]. In humans, a variant of the human klotho gene, the KL-VS allele, has been associated with increased serum klotho levels and with improved cognitive test performance in heterozygote individuals[27]. Increased serum klotho levels have also been associated with reduced risk of cognitive decline[29]

Extremely high serum FGF23 levels have been associated with primary Klotho deficiency (e.g. genetic deletion)[30]. Klotho has been postulated to suppress FGF23 production in the bone[31], thus higher FGF23 levels may reflect lower circulating serum klotho. Alternatively, FGF23 may itself serve to reduce serum klotho levels. Thus, the association between increased FGF23 levels and dementia could be explained by the effects of the anti-aging protein klotho and enhancing the effects of klotho could potentially protect against cognitive decline and dementia.

We did not observe any association between serum FGF23 levels and global neurocognitive test performance, or individual cognitive test performance. However, serum FGF23 levels and neurocognitive test performance were assessed at the same examination cycle and so we were unable to perform a longitudinal analysis. It is possible that elevated serum FGF23 levels may be associated with decline in cognitive test performance over longer-term time follow-up.

The strengths of our study are the inclusion of a population confirmed to be free of clinical dementia at baseline, the use of stringent surveillance procedures for detecting dementia, and inclusion of a large sample of participants with recorded serum FGF23 levels. An important limitation is the inclusion of a predominantly Caucasian population in the Framingham cohort, which may limit the generalizability of our findings to non-Caucasian individuals, as well as the effects of residual confounding due to our inability to adjust for additional measures of mineral metabolism. We were unfortunately unable to measure serum klotho levels to determine if this protein modifies the association between serum FGF23 levels and dementia.

## Conclusions

Higher serum FGF23 was associated with an increased risk of incident dementia, which was not accounted for by increased vascular risk factor burden or vascular measures of structural brain injury. FGF23-related biological pathways may play a role in the development of dementia.
